# Multifunctional nano-in-micro delivery systems for targeted therapy in fundus neovascularization diseases

**DOI:** 10.1186/s12951-024-02614-1

**Published:** 2024-06-20

**Authors:** Xin Liu, Keke Huang, Fuxiao Zhang, Ge Huang, Lu Wang, Guiyu Wu, Hui Ren, Guang Yang, Zhiqing Lin

**Affiliations:** 1 Department of Ophthalmology, The Second People’s Hospital of Chengdu, The Affiliated Hospital of Chengdu Medical College, Chengdu, 610031 China; 2 Department of Ophthalmology, The Third People’s Hospital of Chengdu, The Affiliated Hospital of Southwest Jiaotong University, Chengdu, 610031 China; 3 Department of Ophthalmology, The Third Affiliated Hospital of Chongqing Medical University, Chongqing, 401120 China

**Keywords:** Nano-in-micro (NIM) delivery system, Nanoparticles, Drug delivery, Fundus neovascular disease

## Abstract

Fundus neovascularization diseases are a series of blinding eye diseases that seriously impair vision worldwide. Currently, the means of treating these diseases in clinical practice are continuously evolving and have rapidly revolutionized treatment opinions. However, key issues such as inadequate treatment effectiveness, high rates of recurrence, and poor patient compliance still need to be urgently addressed. Multifunctional nanomedicine can specifically respond to both endogenous and exogenous microenvironments, effectively deliver drugs to specific targets and participate in activities such as biological imaging and the detection of small molecules. Nano-in-micro (NIM) delivery systems such as metal, metal oxide and up-conversion nanoparticles (NPs), quantum dots, and carbon materials, have shown certain advantages in overcoming the presence of physiological barriers within the eyeball and are widely used in the treatment of ophthalmic diseases. Few studies, however, have evaluated the efficacy of NIM delivery systems in treating fundus neovascular diseases (FNDs). The present study describes the main clinical treatment strategies and the adverse events associated with the treatment of FNDs with NIM delivery systems and summarizes the anatomical obstacles that must be overcome. In this review, we wish to highlight the principle of intraocular microenvironment normalization, aiming to provide a more rational approach for designing new NIM delivery systems to treat specific FNDs.

**Graphical Abstract:**

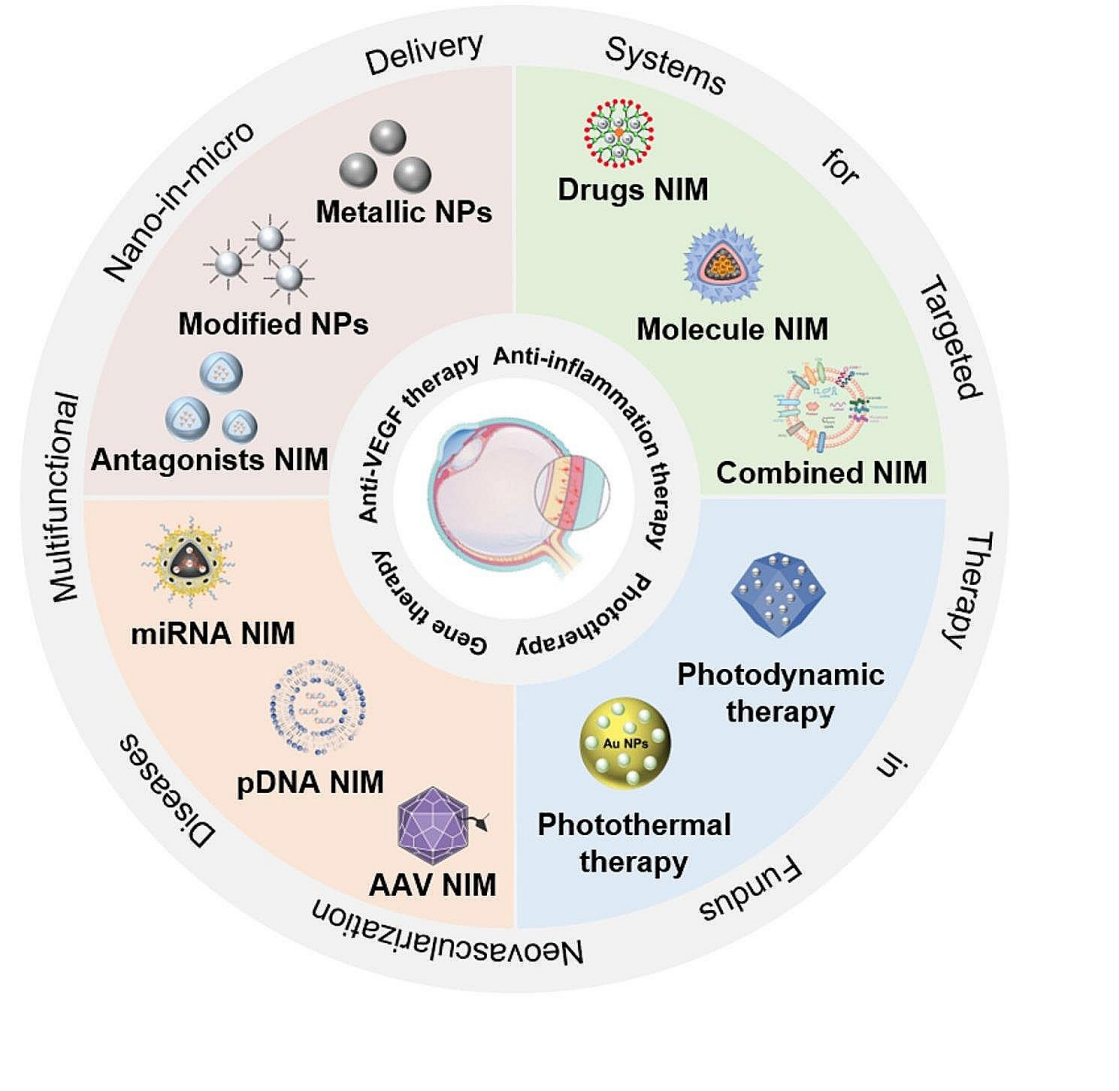

## Introduction

Nano-in-micro (NIM) delivery systems, using both traditional nanomaterials, such as block copolymers, metal nanoparticles (NPs), and some novel nanomaterials, such as up-conversion nanomaterials, metal-organic frameworks, and quantum dots [1–3], are widely used to diagnose and treat cancers, neurodegenerative disease, and eye diseases. Newly developed NIM delivery systems have been shown to deliver drugs or gene editing systems to specific target cell types and ensure their controlled release [4–17]. This, in turn, increases the biological half-life and availability of these drugs, while reducing their toxicity and side effects [9, 18–22].

The intraocular structure of the eye is complex, both anatomically and physiologically. The blood-retinal barrier (BRB) prevents drugs delivered to the eye from spreading to other tissues in the body and reduces systemic adverse effects. Moreover, it is difficult for intraocular delivery methods to breach the limitations of multiple barriers within the eye and overcome the accompanying problems of low bioavailability and short half-life. Various NIM delivery systems have therefore been designed to overcome these barrier restrictions in the treatment of eye diseases. These include NIMs carrying an agent cross-linked by visible light for corneal regeneration [23] and NIMs carrying peptide-drug conjugates (PDCs) with prolonged bioavailability to synergistically treat inflammation associated ocular disorders [24]. Other NIMs consist of polymeric nanocarriers bearing agents with reactive oxygen species (ROS) controlled release [25] and a versatile hybrid hydrogel. These NIMs have been found to exhibit the spatiotemporal properties allowing for drug release [26] in the treatment of infectious keratitis, and gene delivery by solid lipid NPs (SLNs) to treat X-linked juvenile retinoschisis [27]. Several traditional drug delivery strategies and some NIM delivery systems, however, have shown suboptimal efficacy in the treatment of posterior segment retinal disease, largely due to poor targeting ability and the BRB.

This review summarizes the recent developments in NIM delivery systems based on therapeutic strategies for the treatment of fundus neovascular diseases (FNDs), such as NIMs carrying agents that reduce the activity of vascular endothelial growth factor (VEGF), normalized the fundus microenvironment and those that can be used for photodynamic therapy (PDT) and gene therapy. This review also describes recent developments in NIM delivery systems; the features common to different nanomaterials, such as size and surface charge; and their unique biological properties. Lastly, we describe the design of traditional NPs and new NIM delivery systems based on different therapeutic strategies, and their potential clinical applications in the treatment of FNDs.

## Fundus neovascular diseases and their treatment

FNDs are typical posterior ocular diseases, consisting mainly of retinal neovascularization (RNV) and choroidal neovascularization (CNV) [28]. Clinically, the main causes of RNV can be classified primarily as retinopathy of prematurity (ROP), diabetic retinopathy disease (DR), and retinal arteriovenous occlusion (RVO), whereas the causes of CNV mainly refer to wet age-related macular degeneration (wAMD) and high myopia [29]. These diseases usually lead to deterioration of vision and even irreversible blindness. Drug therapy has been a better choice in the treatment of FNDs, on account of conventional laser therapy as the latter can result in loss of vision, a high recurrence rate, and other complications such as scar hyperplasia and secondary choroidal neovascularization [30].

**Fig. 1:**
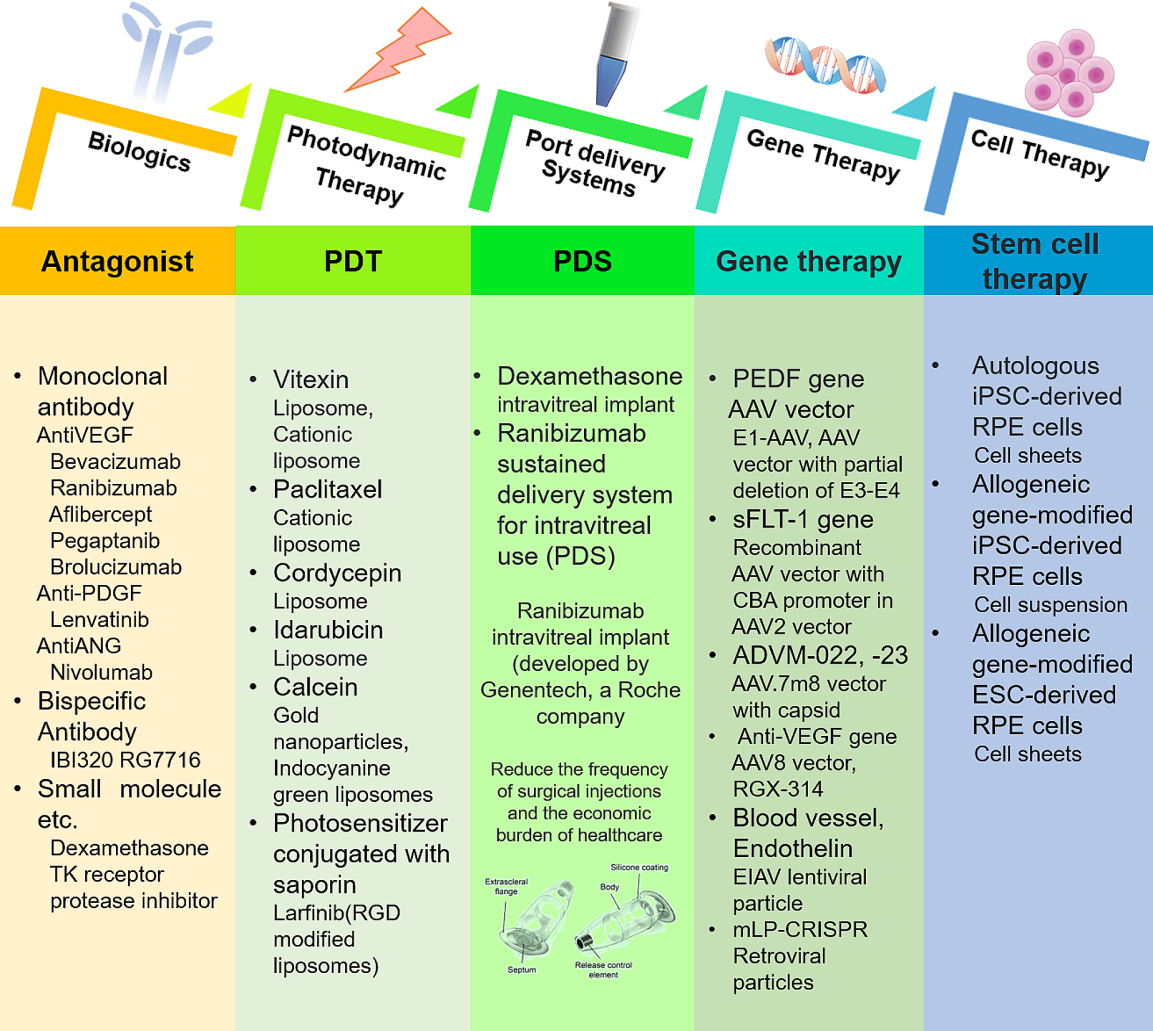
Ocular therapeutics and NIM delivery strategies for FNDs

Preclinical and clinical therapeutic strategies affecting FNDs include anti-VEGF therapy, consisting of bispecific monoclonal antibodies (MAB) and small molecules, PDT, port delivery system (PDS), gene therapy, and stem cell therapy [31]. Relevant ocular therapeutics and NIM delivery strategies for FNDs are shown in Fig. 1. Novel therapeutic strategies have been designed to optimize efficacy, minimize adverse events, and minimize the frequency of interventions [32, 33]. Because VEGF and its receptor, VEGFR, play key roles in the pathogenesis of CNV and RVO [34–37]. Anti-VEGF agents, such as bevacizumab, aflibercept, and ranibizumab can guide the clinical treatment of FNDs. These agents have been shown to markedly reduce the rate of blindness from FNDs worldwide [38–41]. Intravitreal injections of anti-VEGF agents, however, have been linked to ocular complications, systemic side effects, and poor compliance [42].

## Ocular physiologic barriers and nanoparticle-mediated drug delivery across the physiologic barriers

The eye is an organ with complex anatomical and physiological barriers [43]. These complex anatomical and physiological structures of the eye form natural barriers to NPs delivery for treating FNDs [44].

### Different ocular barriers for drug delivery

**Fig. 2:**
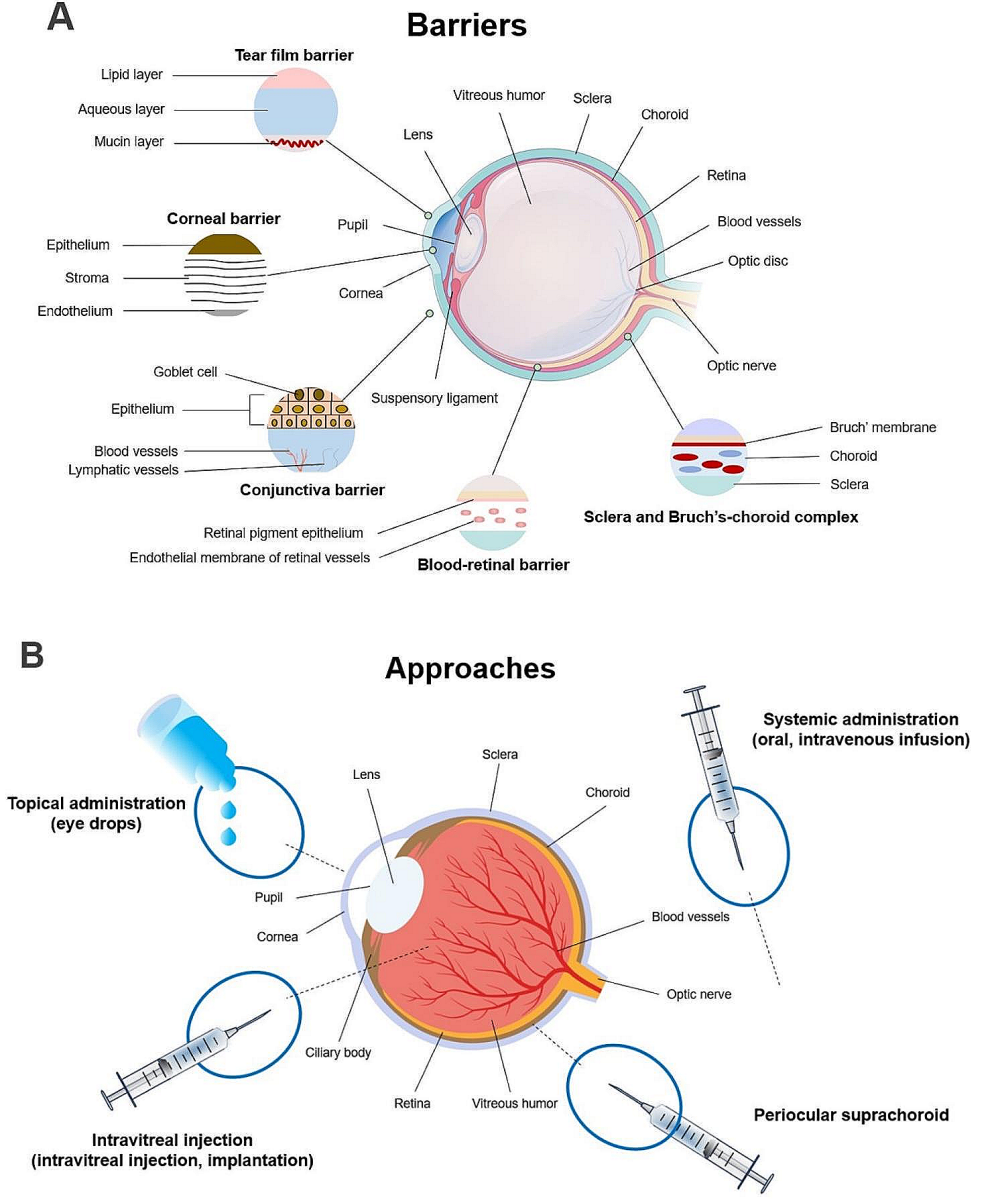
The schematic illustration: **(A)** Physiological barriers in the eye. **(B)** Common methods of drug administration to the eyes

Several physiologic barriers for ocular NPs delivery include tear film barrier, corneal barrier, conjunctiva barrier, sclera and Bruch’s-choroid complex, and blood-retinal barrier [45], as shown in Fig. 2(A).

#### Tear film barrier

The tear film is mainly composed of three layers: the outermost lipid layer, the middle aqueous layer, and the bottom deep mucin layer [46]. Together with the proteins, lipids, and mucins in the tear fluid, they form the dynamic structural barrier [47]. In addition, continuous rapid tear turnover and drainage through the nasolacrimal duct create the dynamic obstacles of the tear film barrier [48]. The dynamic physiological balance of the tear film, the dynamic action of blinking and tear turnover, and the retention and permeability of drug delivery systems on the surface of the eye will become the primary consideration for overcoming the tear film barrier [49, 50].

#### Corneal barrier

The corneal barrier, similar to a sandwich-like structure, is composed of the outermost corneal epithelium, the middle corneal stroma, and the inner corneal endothelium [51]. It is adjacent to the tear film on the outer side and connected to the aqueous humor on the inner side [52]. Due to the composition of different substances in the corneal layers and the expression of various efflux pumps on epithelial and endothelial cells, it forms a barrier that isolates most lipophilic or hydrophilic substances, blocking drug delivery to the posterior segment [53].

#### Conjunctiva barrier

The conjunctiva is a mucous membrane covering the inner surface of the upper and lower eyelids and the front of the eyeball. It is a transparent thin membrane composed of stratified columnar epithelium and a small amount of connective tissue. The conjunctiva lining the inner surface of the eyelids is called the palpebral conjunctiva, and the part covering the eyeball is called the bulbar conjunctiva, with the transition area between them known as the fornix conjunctiva. The conjunctiva contains abundant blood vessels and lymphatics, as well as a few mucous glands that secrete mucus to lubricate the eyeball [54]. However, due to its rich blood vessels and lymphatics that can absorb permeating substances into the systemic circulation, it can result in poor bioavailability during the drug delivery process, leading to significant drug loss. Additionally, the tight junctions between corneal epithelial cells act as a barrier, blocking drug delivery to the posterior segment of the eye [45, 52].

#### Sclera and Bruch’s-choroid complex

As the main component of the eyeball wall, the sclera makes up the posterior 5/6 of the eyeball’s fibrous coat, primarily composed of hydrophilic collagen tissue [52, 55]. Therefore, hydrophilic compounds may diffuse more easily through the proteoglycan aqueous medium in the fibrous stromal pores compared to lipophilic compounds [56]. The membrane is an important structural barrier that can hinder the delivery of local drugs.

The choroid, located between the retina and the sclera, is a thin, soft, smooth, elastic, and highly vascular brown membrane [57]. The choroid is permeable to lipophilic compounds but less permeable to proteins and small hydrophilic substances. Drugs can be transported through both transcellular and paracellular pathways [58]. The Bruch’s membrane anchors the retinal pigment epithelium (RPE) and the choroid, and induces cell polarity and tight junctions [59]. Bruch’s membrane is semi-permeable and has a high negative charge due to the presence of proteoglycans, giving it a function similar to an io exchange column [59]. Bruch’s membrane is constantly renewed, with matrix metalloproteinases (MMPs) degrading the Bruch’s membrane and being replenished by the choroid, which is crucial for maintaining the barrier function of Bruch’s membrane. The complex composed of the choroid and Bruch’s membrane can serve as a permeation barrier for drug delivery to the back of the eye [60].

#### Blood-retinal barrier

The blood-retina barrier is an important component of retinal tissue physiology, consisting of retinal blood vessels and the retinal pigment epithelium. The blood-retina barrier consists of an inner barrier and an outer barrier [61].

The inner barrier is the endothelial barrier, composed of retinal capillary endothelial (RCE) cells and their tight junctions. Retinal capillaries have strict selective permeability, with adherens junctions and occluding junctions connecting between endothelial cells, with the latter located near the lumen and the former being the main connecting part. The RCE has poor permeability to proteins and small hydrophilic drugs [62]. The outer barrier is the epithelial barrier, consisting of retinal pigment epithelium (RPE) cells and their junctional complexes. The connections between cells include gap junctions at the apical side, occluding junctions in the middle, and adherens junctions at the basal side. Some lipophilic substances can passively permeate RPE to reach the posterior segment [63]. The blood-retina barrier plays a crucial role in limiting the diffusion of drugs from the vitreous humor to the retina.

### Nanoparticle-mediated drug delivery

The interactions and biological properties of NPs are dependent on their morphology, dimensions, and other physical and chemical properties [64–67]. For example, positively charged NPs readily pass through cell membranes to facilitate the intracellular delivery of drugs, NPs with a size of approximately 200 nm can easily pass through the BRB [68]. The designs of NPs targeting FNDs are based on their methods of ocular administration, as they must cross the physiological barriers of ocular and achieve high drug concentrations at neovascularization. Common routes of drug administration include topical ocular administration, vitreous cavity, suprachoroidal cavity, and systemic injection [48, 50, 69, 70]. NPs for specific drug delivery methods may have similar physical and chemical properties although the nanomaterials themselves may vary [71–73]. Different drug administration approaches are shown in Fig. 2(B).

#### Noninvasive drug delivery routes

Eye drops, as a non-invasive and convenient method of topical ocular drug administration, require drugs to overcome the anatomical barrier of the cornea and the physiological barrier of tears, resulting in low bioavailability [69, 74]. Tear film, which is composed of water, mucin, and oils, covers and fills the corneal surface to prevent foreign body invasion. The presence of tear film provides the corneal epithelium with a negative charge at physiological pH value [75]. In addition, the cornea, a highly ordered tissue consisting of three layers, the epithelium, stroma, and endothelium plays an indispensable role in protecting the eye from foreign bodies [76]. Due to the tight connections between epithelial cells, lipophilic substances must pass through the epithelium via the cross-cell pathway, and the corneal endothelium shares similar characteristics with the epithelium [77]. The corneal stroma is highly hydrophilic, making hydrophilic substances easily diffusible within the corneal stroma [78]. Therefore, positively charged amphiphilic drug carriers usually have better corneal permeability than the neutral or negatively charged single hydrophilic drug carriers, suggesting that the former are better for drug delivery and provide improved bioavailability in the treatment of the FNDs [79]. Positively charged substances, however, can disrupt cell membranes and cause toxicity, suggesting the need to pay more attention to the biological safety [80–83]. In addition to the hydrophilicity and charges of drug delivery systems, their efficacy is also dependent on their biocompatibility with tear film and their adhesive properties, with these characteristics being important considerations in the formulation of eye drops [84]. Research on NIM delivery Systems for topical delivery has shown progress with hydrogels, cyclodextrin, permeable polypeptide-modified amphiphilic compounds, lipid nanoparticles (LNPs), and others being tested [85–90]. These systems can significantly improve corneal retention time, drug delivery efficiency, and bioavailability.

#### Invasive drug delivery routes

Drugs administered by intravitreal and suprachoroidal injections, must overcome the barriers provided by the vitreous, Bruch’s membrane, and retinal pigmented epithelial (RPE) cells layer [45]. Therefore, the dimensions and surface properties of NPs play a key role in drug delivery and fundus-specific cell targeting. The vitreous humor is composed of 98–99% aqueous substance and 1–2% collagen and hyaluronic acid (HA). Positively charged NPs are more likely to bind to negatively charged HA, hindering the diffusion of these NPs, whereas, negatively charged NPs are more likely to spread to the deeper retina [91–93]. The migration and diffusion efficiency of NPs in the gel-like vitreous cavity differs depending on their composition and size. Polystyrene (PS) NPs 510 nm in size were found to be able to rapidly diffuse to the retina, whereas the diffusion rate of materials larger than 1 μm in the vitreous cavity is much more limited. NPs about 100 nm in size are more likely to overcome resistance of the vitreous structure and have confirmed an interaction with Müller cells. Although small-sized NPs have a high diffusion rate, they are generally unable to a sufficient drug concentration in the retina and show a sufficient therapeutic effect because of their short residence time, ready metabolism, and the influence of eye movement [92, 94, 95].

The BRB consists of tight junctions between retinal endothelial cells and RPE cells [61, 96]. Due to this limitation, macromolecules and NPs > 2 μm in diameter cannot effectivity penetrate the deep retina [97], The permeability of the BRB to small molecules depends on their lipophilicity [70, 98]. Because systemically administered NPs are inevitably deposited in other organs, their potential biosafety should be prioritized, including their biodistribution, clearance, side effects, and toxicity. Tropically administered NPs can successfully infiltrate into the retina, making it difficult to specifically target the diseased cells or unmodified sites. Intelligent nanomaterials can therefore be designed by modifying the surfaces of NPs with aptamers to target specific cells and treat patients more precisely [99, 100].

## In vitro and in vivo evaluation of NIM delivery systems targeting FNDs

The safety and effectiveness of the intelligent NIM delivery systems have been evaluated in cell culture and eye organ models in vitro and animal models. New Zealand white rabbits are the most common animal models because they are inexpensive, easy to obtain, and have been used in international standard tests evaluating the acute toxicity of ophthalmic drugs [101, 102]. The primary sources of in vitro models of ocular organs are isolated rabbit, chicken, bovine, and porcine eyes. Animal models of FNDs include choroidal neovascularization (CNV), oxygen-induced retinopathy (OIR), diabetic retinopathy (DR), and retinal vein occlusion (RVO). In vitro cell culture models include human RPE cells (ARPE-19), human retinal microvascular endothelium cells (HRMECs), and retinal capillary endothelium cells (HRCECs). In vitro cell and animal models are rapid and easy to construct, have a short-cycle, and are cost-effective. These models are therefore essential for characterizing the physical and chemical properties of intelligent NIM delivery Systems including particle size, morphology, stability, sterility, permeability, the efficiency of drug release, and in vitro cellular biocompatibility and toxicity [103]. Table 1 summarizes the in vitro and in vivo evaluation models for NIM delivery systems targeting FNDs Table 2.

**Table 1:** Listing of in vitro cell culture models, animal models, and organ models of FNDs

Cell culture model in vitro	Name	Species	Application
Cell type	ARPE-19	Human	Morphology, stability, sterility, drug release time, and efficiency
	HRMEC	Human	
	HRCEC	Human	

Fundus neovascular animal model in vivo	Animal model	Species	Inducing method
Choroidal neovascularization model	Laser-induced CNV model	Rat, mouse, rhesus	Argon laser, nitrogen red laser
	Operation Induced CNV model	Rat	Subretinal injection of synthetic peptides, cells, and adenovirus vector containing VEGF gene
Retinopathy of prematurity model	OIR model	Newborn mouse	Mice were exposed to 75% hyperoxia on the seventh day after birth and then to normal air for another 5 days
Diabetic retinopathy animal model	Spontaneous hereditary diabetes mellitus model	Rat	Inbreeding, genetic testing
	STZ-induced diabetic retinopathy model	Rat	Streptozocin (STZ) chemical damage
Retinal vein occlusion model	Induced retinal vein occlusion model	Rabbit	Ammonia laser photocoagulation, condensation, thrombin intravenous drip, intravitreal injection, and other methods

Ex vivo **organ models**	**Name**	**Species**	**Application**
Eye	IRE	Rabbit	Drug stability, permeability, drug release, and metabolism time, toxicity, irritation
Eye, embryo	ICE, Chorioallantoic membrane	Chicken	
Eye	IBE	Cow	
Eye	IPE	Pig	

**Table 2:** Listing of delivery strategies for FNDs

Therapeutic Modality	Active Ingredient	Delivery System	Highlights	Refs.
Metallic NPs	AuNPs	Intravenous injection	Significantly decreased fluorescein leakage at the CNV site and reduced tissue damage	[119]
	AuNPs	Polyethylene glycol modified gold nanorods	Targeting, magnetic imaging, ferroptosis induction, and immunotherapeutic properties	[121]
	Iron oxide NPs	Iron oxide nanoparticles incorporated with exosomes (ESIONPs@Exo)	Bionic NPs based on hybrid cell membranes for non-invasive targeted therapy of CNV	[122]
Modified NPs	Bionic NPs	Intravenous injection	Bionic NPs based on hybrid cell membranes for non-invasive targeted therapy of CNV	[123]
	Anti-VEGF aptamer	Hybrid carbon dots	Sustained release and noninvasive monitoring intraocular concentration	[124]
Antagonists NPs	Bevacizumab	Mesoporous silica NPs	Prolong the residency of drug in vitreous and maintain the long-lasting drug concentration	[131]
	Bevacizumab	Poly (lactide-co-glycolide) (PLGA) NPs	Increase the bioavailability and decrease the toxicity of bevacizumab	[284], [285]
	Bevacizumab	PLGA/poly(cyclohexane-1,4-diyl acetone dimethylene ketal) (PLGA/PCADK) microspheres	Highly bioactive and sustained-release	[132]
	Bevacizumab	Graphene quantum dots (GQDs) encapsulated with supramolecular β-cyclodextrin (β-CD)	High level, mass transfer, structural control, high compatibility, and degradation nature	[134]
	Bevacizumab	Chitosan-coated PLGA NPs	Sustained release	[135]
	Bevacizumab	Chitosan nanoparticles	Transport, targeting, and controlled release of drugs	[136]
	Bevacizumab	Chitosan grafted-poly (ethylene glycol) methacrylate NPs	Minimally invasive and sustained release	[137]
	Ranibizumab	PLGA based NPs-loaded bilayer microneedles	Minimally invasive and sustained release	[138]
	Bevacizumab	Multivesicular liposomes	Highly biocompatibility and sustained release	[139]
	Ranibizumab	RGD peptide-targeted Core Cross-linked Star (CCS) Polymers modified with indocyanine green	Highly biocompatibility and sustained-release	[141]
	Pazopanib	High-density lipoprotein (HDL) mutant	The better absorption of drugs by cells and penetration	[142]
	Sunitinib	Microparticles (MPs) composed of blends of PLGA and PLGA conjugated to polyethylene glycol (PLGA-PEG)	Sustained release, elimination of ocular inflammation, and reduced dispersion of MPs into the visual axis.	[143]
	Aflibercept	Copolymer EPC (nEPCs)	Highly biocompatibility and sustained-release	[144]
	Connexin43 mimetic peptide (Cx43MP)	PLGA NPs	Highly biocompatibility and sustained-release	[148]
	KV11 peptide	Exosomes	Efficient and less invasive	[152]
Drugs/molecule NPs	Dexamethasone	PLGA and N-methyl-pyrrolidin (NMP)	Monitoring of dynamic changes in the implants, controlled release of drugs	[172]
	Dexamethasone	PLGA NPs	Highly biocompatibility and sustained release	[173]
	Triamcinolone acetonide (TA)	TA-loaded solid lipid NPs and in situ gel (TA-SLN-IG)	Improved permeability, highly biocompatibility, and sustained release	[174]
	Triamcinolone acetonide (TA)	Systemic hydroxyl-terminated polyamidoamine dendrimer-triamcinolone acetonide conjugate (D-TA)	Hydroxyl PAMAM dendrimers target reactive microglia/macrophages and hypertrophic RPE	[175]
	Resveratrol	AuNPs	AuNPs therapy inhibits ERK1/2 signaling pathways and improves retinal inflammation by trans inhibiting NF-κB	[177]
	Oxidant salvianolic acid A (SAA)	Arginine-glycine-aspartic acid (RGD)-polyethyleneimine (PEI)	Desirable water dispersibility, low cytotoxicity, and sustainable release of drugs	[178]
	Fenofibrate	PLGA NPs	Highly biocompatibility and sustained-release	[180]
	Triptolide	Liposomes modified with the peptide APRPG (Ala-Pro-Arg-Pro-Gly)	Highly biocompatibility and sustained release	[181]
	Diclofenac	Liposomes	Highly biocompatibility and sustained-release	[182]
	Atorvastatin	Solid lipid NPs	Statins are beneficial for the control of macular degeneration and are effective in high drug concentrations	[183]
	Doxorubicin	PEG-PLA chains modified with a cell penetrating	Light triggered targeting and controlled drug release	[185]
	Doxorubicin	MRP@DOX co-assemble nanoparticles consisting of glycopeptide, cationic peptide, and DOX	Good histocompatibility, non-invasive treatment, and targeting M2 macrophages in the fundus	[186]
Combined NIM	Bevacizumab and dexamethasone	PLGA Polyethyleneimine (PEI) NPs (eBev-DPPNs)	The combination of steroids and anti-VEGF drugs has advantages in the treatment of CNV	[199]
	Bevacizumab and dexamethasone	cRGD peptide-targeted NPs of bevacizumab and dexamethasone with (aBev/cRGD-DPPNs)	aBev/cRGD-DPPN was selective to αVβ3 overexpressed ARPE-19	[200]
	Bevacizumab and triamcinolone acetonide	Hybrid lipid NPs	The incorporation of lipophilic and hydrophilic therapeutic molecules in the same nanoparticle	[201]
	Nintedanib and lutein	Co-assemble into NPs	Sustained release	[202]
	Anti-VEGF antibodies (aV) and peptide linker (cL)	Exosomes derived from regulatory T (Treg) cells (rEXS)	Engineered exosome targeted accumulation in neovascularization	[209]
	Bevacizumab and graphene oxide quantum dots (GOQDs)	Chemically bonding GOQDs to an octadecyl-modified peptide sequence (C18-GVFHQTVS, C18P)	MMP9-responsive, macrophage/microglial inhibitor, anti-inflammatory action, and antiangiogenic effects	[210]
Photothermal therapy	Bevacizumab	Polymer-coated AuNPs inside an agarose hydrogel	Photothermal effects soften hydrogels to control the release of drugs	[223]
Photodynamic therapy	Indocyanine green (ICG)	AuNPs	The ICG-associated enhancement of AuNPs permeability and persistence can assist in targeting CNV	[228]
	Indocyanine green (ICG)	PtNPs and AuNPs decorated with a zeolitic imidazolate framework-8 nanoplatform	Targeting of the CNV, precise PDT occlusion of neovascular vessels	[4]
	Verteporfin (VER)	Photoactivatable nanosystem (Di-DAS-VER NPs)	Light triggerd sustained and controlled drug release	[230]
Non-viral vectors	siRNA-based anti-VEGF	Polyethyleneimine (PEI) and hyaluronic acid (HA) nanosphere	Targeting CD44 receptors and sustained release	[245]
	VEGFR-2 siRNA	Chitosan-hyaluronic acid nano-polyplexes	Low toxicity without considerable immunologic reactions	[246]
	MicroRNA-146a-5p inhibitor	APRPG modified PEG nanoliposomes	Targeting neovascularization	[247]
	MicroRNA-23 antisense oligonucleotides	Cowpea chlorotic mottle virus-like particle (CCMV-VLP) was cross-linked to anti-microRNA-23 by 3,3’ Dithiobis (sulfosuccinimidyl propionate)	Targeting microRNA-23	[248]
	MicroRNA-223	Folic acid–chitosan (FA–CS)-PEG-modified mesoporous silica NPs (FACS/PMSN)	Targeting microglial and driving microglial polarization toward the anti-inflammatory phenotype	[249]
	Plasmid Flt23k	RGD-targeted peptide-modified NPs	Neither toxicity nor inflammation	[250]
	Plasmid anti-VEGF	RGD-targeted peptide-modified NPs	Neither toxicity nor inflammation	[251]
	Vascular cell adhesion molecule-1 (VCAM-1)	AuNPs functionalized with hairpin-DNA that incorporates an anti-sense sequence complementary	Systemic delivery of AuNPs to ocular tissues to facilitate mRNA imaging of ocular disease models	[252]
	plasmid SOX9 DNA	Sunflower typed nanoparticles (SF-NPs)	Stem cells that internalized nanoparticles at the early stage retained genetic stability, even after passage	[253]
	RNPs targeting VEGF	Nano-capsules (NCs) consisting of a thin covalently-crosslinked glutathione polymer	Effective targeted gene editing capabilities	[257]
Viral vectors	SpCas9	Lentivirus vectors (LVs)	Targeting exon 3 instead of exon 1, achieved VEGF-A knockout	[262]
	CjCas9	AAV9	The type V (class II) CRISPR and Francisella 1 catalyzes staggered DNA cleavage	[263]
	SpCas9 and gRNAs	AAV8	Higher targeted editing rates	[264]
	CasRx (RFXCAS13D)	AAV	Targeting of VEGF-A mRNA	[265]
	Cas9-mRNA and a gRNA	Engineered lentivirus	Targeting of VEGF-A mRNA	[267]
	SpCas9 and ICAM2	AAV5	Effectively knock out VEGFR-2	[268]
	SpCas9	Lentiviral vectors	Effectively knock out VEGFR-2	[269]

## NIM delivery systems based on different therapeutic strategies for FNDs

### Anti-VEGF systems

Blood vessels are formed by the process of endothelial and smooth muscle cells interacting with each other [104]. Fundus neovascularization includes the differentiation and development of retinal endothelial cells and the formation of a vascular network, a complex process, regulated by various cytokines and signaling pathways [105, 106]. Among them, local overexpression of VEGF is a key pathological feature of retinal neovascularization. In clinical practice, anti-VEGF can effectively inhibit the formation of various types of neovascularization in the retina, thereby delaying disease progression. However, long-term use of anti-VEGF drugs may lead to the development of resistance, reducing the efficacy of the medication [107]. Additionally, due to its pharmacokinetic characteristics and bioavailability, anti-VEGF therapy often requires frequent injections, imposing a significant financial burden on patients [108]. Therefore, to overcome these obstacles, a large number of novel and effective NIM delivery systems have been developed to enhance the efficacy of anti-VEGF therapy, but many NPs have potentially toxic effects on organisms. These side effects may be caused by oxidative stress, response to inflammatory stress, and genetic damage, thus, various NIM delivery systems are being designed to effectively deliver drugs or NPs associated with anti-VEGF intraocularly, ensuring the safety of drugs or NPs for in vivo treatment is essential in the design of NIM delivery systems [109–111].

#### Metallic nanoparticles targeting VEGF

Some nanomaterials have been reported to inhibit angiogenesis, whereas gold, iron oxide, cerium oxide, titanium dioxide, and silica NPs have been reported to resist fundus neovascularization [112–117]. Gold NPs (AuNPs) widely used in the diagnosis and treatment of FNDs were shown to inhibit VEGF-induced endothelial cell migration by inhibiting the Akt/eNOS phosphorylation pathway without affecting the normal physiological adhesion of choroidal-retinal endothelial cells to fibronectin (Chen et al.) [118]. Optical coherence tomography (OCT) and fundus fluorescein angiography (FFA) imaging show that whole-body injection of AuNPs significantly inhibited laser-induced choroidal neovascularization in mice (Rupesh Singh et al.) [119]. Moreover, AuNPs can inhibit hypoxia-induced retinal neovascularization by inhibiting autophagy [120]. Polyethylene glycol-modified gold nanorods were shown to act as potential novel cytokinesis inhibitors by inhibiting angiogenesis in an OIR mouse model (Song et al.). The mechanism of action of these nanorods was associated with their inhibition of the transforming growth factor pathway, which in turn affects actin self-assembly [121]. Iron oxide nanoparticles can suppress pathological neovascularization by inducing ferroptosis. Therefore, by sorting the exosomes secreted by ESIONP-induced M1 polarized macrophages, extremely small iron oxide nanoparticles (ESIONPs) incorporated with exosomes (ESIONPs@Exo) can be obtained (Song et al.). This method, using macrophages as bioreactors, produces exosomes with targeting, magnetic imaging, ferroptosis induction, and immunotherapeutic properties, demonstrating the potential for targeted therapy of pathological neovascularization [122].

#### Modified nanoparticles targeting VEGF

**Fig. 3:**
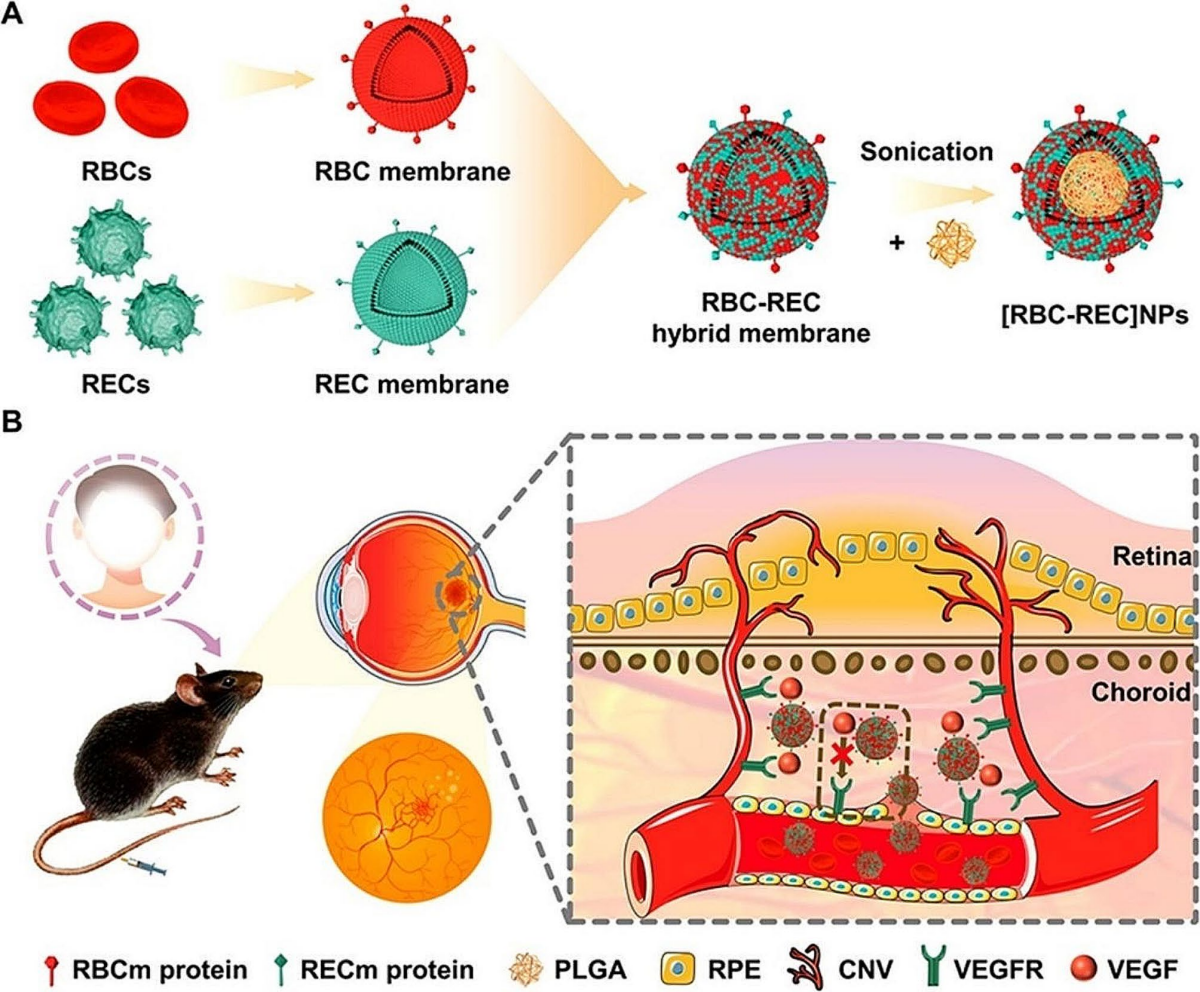
Schematic depiction of hybrid cell membrane-coated biomimetic nanoparticles intended for non-invasive targeted therapy of laser-induced CNV. **(A)** Process of preparing [RBC-REC]NPs by encapsulating polymeric cores with integrated RBC-REC membranes. **(B)** Capture of proangiogenic factors by intravenous administration of [RBC-REC]NPs, inhibiting the effects of these factors on host neovascular endothelial cells

Some modified NPs can competitively bind to VEGF receptors, replacing anti-VEGF drugs in the treatment of FNDs. And cell-membrane-derived biomimetic nanoparticles have shown promising applications in various biomedical fields, particularly in drug delivery. This led to the development of bionic NPs based on hybrid cell membranes for non-invasive targeted therapy of CNV (Su et al.) (Fig. 3). The mixed cell membrane-coated nanoparticles constructed by fusing red blood cell (RBC) membranes with retinal endothelial cell (REC) membranes ([RBC-REC] NPs) can autonomously recognize and target retinal endothelial cells upon entering the body via the intravenous delivery. The CD47 expressed on the red blood cell membrane provides immune evasion in the circulatory system. The nanoparticles derived from retinal endothelial cell membranes, containing VEGFR2, can absorb the VEGF-A ligand. These biomimetic antiangiogenic NPs can reduce the leakage and area of CNV [123]. Similarly, hybrid carbon dots functionalized with the VEGF aptamer were found to effectively inhibit choroidal angiogenesis, with an activity comparable to the activities of bevacizumab and aflibercept (Shoval et al.). The intrinsic fluorescence of carbon quantum dots can also be used to noninvasively monitor intraocular concentrations [124]. Ultimately, these modified nanoparticles targeting VEGF provide an effective alternative that meets the need for non-invasive treatment of FNDs.

#### VEGF antagonists

**Fig. 4:**
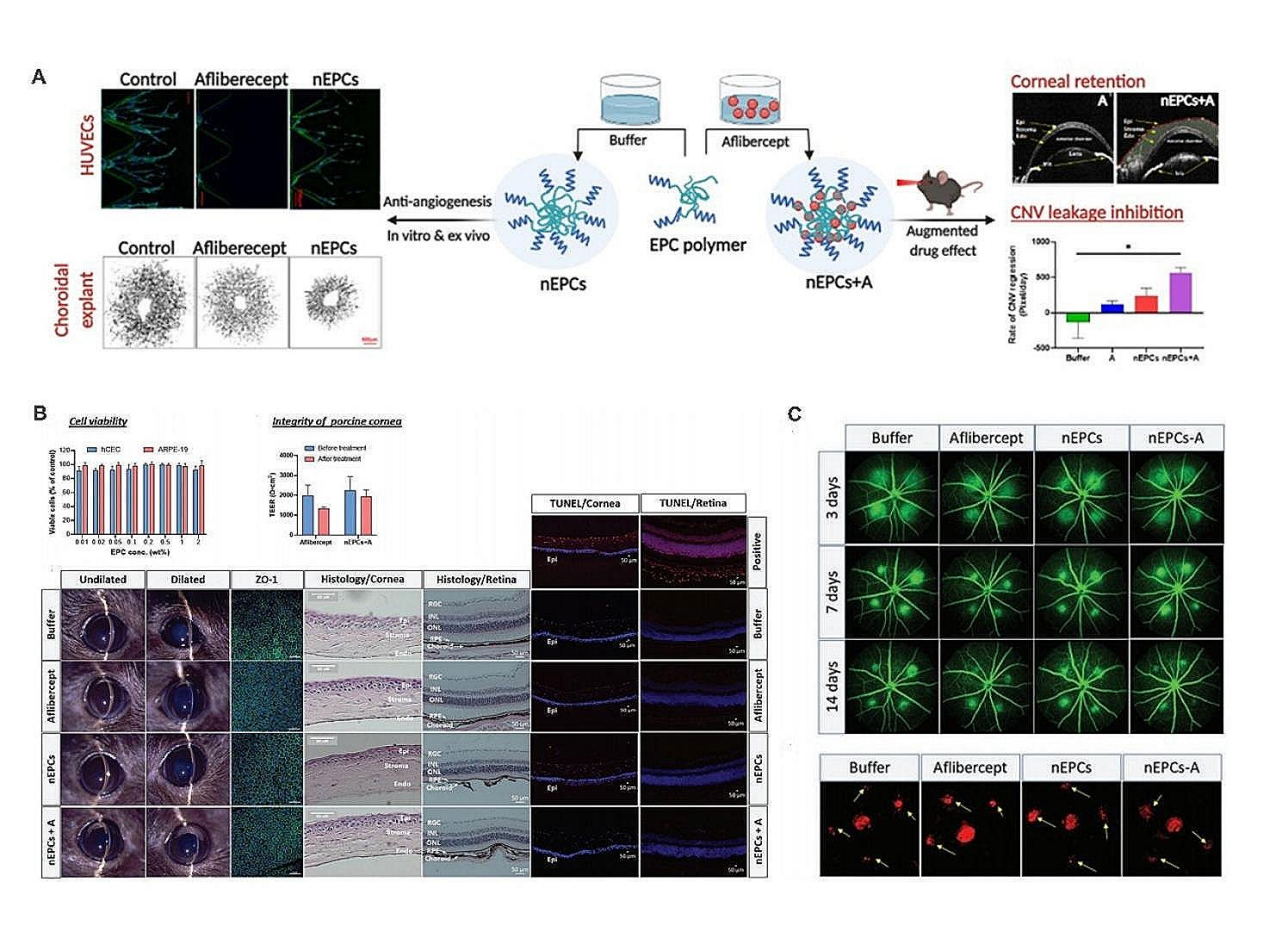
A nanomicelle drug delivery system composed of copolymer EPC (nEPCs) carrying aflibercept for the treatment of FNDs. **(A)** Schematic diagram of aflibercept loaded nEPCs administratered ex vivo porcine eye were able to penetrate the cornea and effectively deliver a clinically relevant dose of aflibercept to the retinas with laser-induced choroidal neovascularization (CNV), resulting in regression of CNV. **(B)** Schematic diagram of biocompatibility of nEPCs in human cornea and RPE cell lines, in porcine corneal tissue, and in mice models. **(C)** Schematic diagram of the fluorescence leakage degree in choroidal lesion area and the staining of endothelial cells on the choroidal flat mounts

Many angiogenic factors are involved in the regeneration of blood vessels, including VEGF, platelet-derived growth factor (PDGF), transforming growth factor (TGF), and hypoxia-inducible factor 1 (Hif-1). VEGF considered the most critical angiogenic factor in the pathogenesis of FNDs [34, 125, 126], is a cytokine secreted by endothelial cells and is responsible for the formation of new blood vessels. Serum concentrations of VEGF protein are high in patients with and without FNDs, suggesting that VEGF overexpression is related to the occurrence and progression of FNDs [127]. The U.S. Food and Drug Administration has approved the use of VEGF antagonists such as ranibizumab, and aflibercept [128–130]. NIM delivery systems for these VEGF antagonists have been designed to reduce the need for repeated injections and side effects of treatment. The bevacizumab-loaded NPs for fundus delivery and the treatment of CNV and RNV include AuNPs, silica NPs [131], poly(lactide-*co*-glycolide) (PLGA), poly(D, L-lactide-co-glycolide)/poly(cyclohexane-1,4-dial acetone dimethylene ketal) (PLGA/PCADK) [132], β-cyclodextrin [133, 134], chitosan-coated PLGA (CS-PLGA) [135], chitosan NPs embedded in hyaluronic acid implant [136], chitosan grafted-poly(ethylene glycol) methacrylate NPs [137], nanoparticle-loaded microneedle (NM) arrays [138], multivesicular liposomes (MVLS) [139] and MMP9-responsive graphene oxide quantum dots (GOQDs) [140]. NIM delivery systems composed of graphene quantum dots (GQDs) and β-cyclodextrin were developed for the delivery of bevacizumab and ranibizumab, respectively (Qian et al.) [134]. A system composed of indocyanine green (ICG) and targeted peptide RGD-modified core cross-linked star (CCS) polymer was developed for the delivery of VEGF antagonists to fundus angiography and CNV areas by systemic injection (Yu et al.) [141]. The combination of a cell-penetrating peptide (CPP) and a therapeutically active high-density lipoprotein (HDL) was developed to deliver the anti-angiogenesis drug pazopanib (Kenji Suda et al.). These CPP-fused HDL nanoparticles were found to penetrate the cornea to the deep retina in the treatment of AMD [142]. Sunitinib was reported to self-assemble with poly (lactic-co-glycolic) acid (PLGA) and PLGA conjugated to polyethylene glycol (PLGA-PEG) from sunitinib microparticles (Hiroki Tsujinaka et al.). The PEG coating on the microparticle (MPs) can effectively reduce the risk of inflammation or IOP caused by frequent intraocular injections, and the encapsulation of sunitinib in PLGA-PEG and PLGA polymers can effectively enhance the biocompatibility of sunitinib MPs with ocular tissues. More importantly, the modified sunitinib MPs can aggregate in the vitreous cavity after intravitreal injection, forming a depot that degrades over time and gradually releases sunitinib. This effectively reduces side effects such as visual axis obstruction and vision impairment caused by the dispersion of MPs after injection. A single intravitreal injection of these polymers resulted in sustained inhibition of VEGF signaling and blocked VEGF-induced leukostasis and retinal non-perfusion [143]. Similar microspheres were also developed for apatinib delivery. A nano-micellar protein drug delivery system made of copolymer EPC (nEPC) was found to deliver aflibercept to the posterior segment of the eye via the horn-sclera pathway (Zhao et al.) (Fig. 4). EPC consists of polyethylene glycol (PEG), polypropylene glycol (PPG) and polycaprolactone (PCL) segments. The NIM delivery system can penetrate the cornea of porcine ocular organs in vitro and deliver aflibercept to the deep retina allowing treatment of CNV in an AMD model. In addition, nEPC has intrinsic anti-angiogenic properties and synergizes with aflibercept to exert anti-neovascularization effects, providing a new therapeutic strategy for FNDs [144]. In addition to proteins, peptides have shown promise in the treatment of FNDs, and several NIM delivery strategies have been designed to improve their bioavailability [145]. Connexin43 mimetic peptide (Cx43MP) can be used to treat various retinal inflammations [146]. It is essential to develop a safe, effective, and low side-effect nano-delivery system for carrying Cx43MP to the posterior segment of the eye. As is well known, PLGA is a biocompatible polymer that can hydrolyze into non-toxic oligomers and monomers of lactic and glycolic acid [147]. The preparation of polylactic-co-glycolic acid nanoparticles (PLGA NPs) was maximized using a nano-precipitation technique (Rohit et al.). Then, the Box-Behnken design (BBD) and response surface methodology (RSM) were used for multivariate statistical analysis to evaluate the drug-polymer interactions, testing their compatibility and the suitability of the formulation development. This optimized NIM delivery system was found to be an effective and sustained method for Cx43MP delivery to retinal tissues, which will reduce the need for frequent intravitreal injections, minimize any associated risks, and maintain the required drug therapy concentration in the target organ for an extended period [148]. Exosomes (Exo) have also shown great advantages in FNDs treatment. Exosomes are small vesicles (50–150 nm) containing complex RNA and proteins that arise primarily from polyvesicles formed by intracellular invagination of lysosomal microparticles, the polyvesicles were fused with the cell membrane and released into the extracellular matrix [149]. Exo are low immunogenicity and high safety profile [150]. By manipulating them in vitro, loading specific cargo (such as siRNA, miRNA, peptide/protein, and drugs), and then delivering them to target cells for therapeutic or bioengineering purposes [151]. For example, the use of Exo as a carrier greatly enhanced the antiangiogenic effect of the KV11 peptide delivered by retro-orbital injection (Hua et al.). Furthermore, considering the natural homing properties of Exos, this study chose endothelial cells (ECs) derived Exos as carriers for vascular delivery because Exos derived from ECs have been shown to more effectively home to vascular endothelium for treating FNDs compared to Exos secreted by other cells [152].

### Normalization of the fundus microenvironment

Compared to anti-VEGF treatment methods which aim to directly reduce the production of VEGF in vascular endothelial cells thereby inhibiting fundus neovascularization, normalization strategies that aim to normalize the fundus microenvironment selectively reverse the key molecular signals for pathological neovascularization. These do not damage normal retinal cells and do not increase the risk of inhibiting physiological vascular formation, thereby achieving a long-term effective anti-pathological neovascularization effect.

Although the molecular mechanisms underlying FNDs development remain unclear, extracellular matrix (ECM) homeostasis and immunity dysfunction are thought to be involved in pathological fundus neovascularization [153]. Clinical and preclinical studies have highlighted the roles of microglia and macrophages in disease progression and neovascularization [154–165]. Anti-VEGF drugs can effectively control neovascularization, but can’t resolve inflammation and endogenous VEGF production. Moreover, long-term inhibition of VEGF may affect the development of physiological blood vessels, therefore the use of anti-VEGF treatment has not been widely clinically approved for use in ROP worldwide. Steroid drugs such as dexamethasone and triamcinolone acetonide are anti-inflammatory drugs widely used to treat FNDs [166–168], however, due to theirs long-term vitreous cavity drug release, they will inevitably significantly increase the risk of elevated intraocular pressure and cataracts, which greatly limits its large-scale clinical application [169, 170]. Additionally, oxidative stress system dysfunction and distorted immune regulation may also promote pathological neovascularization. Through highlighting such overlaps in the fundus microenvironment, this part aims to illuminate how to design relevant NIM delivery systems to target the normalization of the fundus microenvironment in FNDs.

#### Anti-inflammatory and anti-oxidative stress drugs

**Fig. 5:**
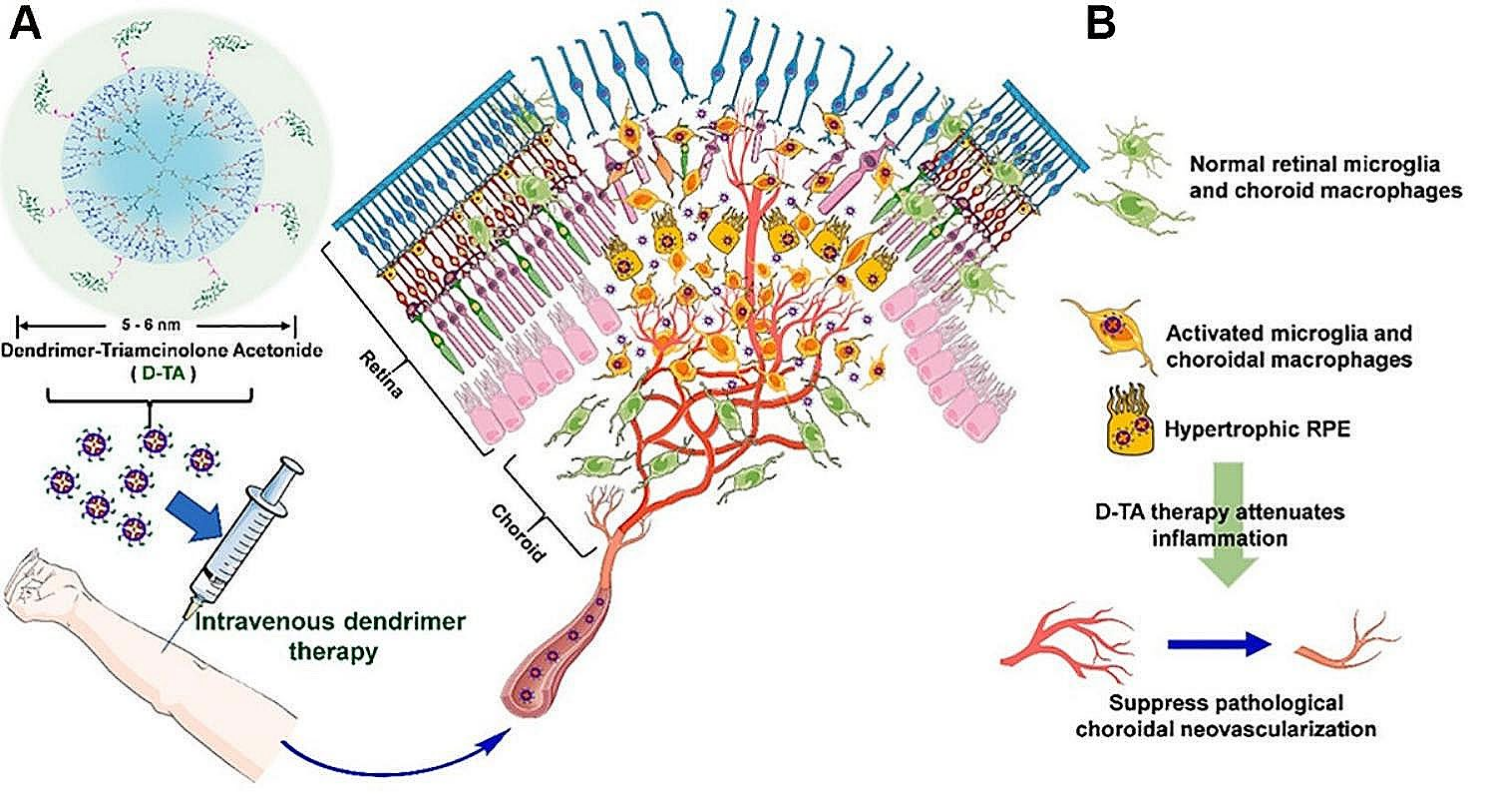
An illustrated outline of the systemic, precise delivery of dendrimer triamcinolone acetonide (D-TA) to treat AMD. **(A)** Intravenously administered. D-TA was found to specifically target and transports D-TA to the crucial cells involved in the progression of AMD. **(B)** This targeted drug delivery approach could significantly reduce inflammation and choroidal neovascularization, ultimately decelerating the progression of the disease

Inflammation is a biological process where the innate immune system activates immune and non-immune cells to combat pathogens. A normal inflammatory response can defend against external invaders such as bacteria, viruses, and toxins. However, if harmful substances persist, acute inflammation may not resolve and instead progress into chronic persistent inflammation, which can have long-term effects on our bodies. ROS are well-known inflammatory mediators that are produced in small amounts in normal human tissues but are generated in large quantities at sites of inflammation. Oxidative stress caused by the overexpression of ROS is a key feature of inflammatory diseases [171]. Inflammation and oxidative stress are the two most important therapeutic targets for normalizing the retinal microenvironment. A series of NIM delivery systems have been designed for the delivery of anti-inflammatory and anti-oxidative stress drugs. For example, several types of PLGA and N-methyl-pyrrolidine (NMP) forming implants in situ have been developed for the controlled release of dexamethasone in the eye (Bode et al.) [172]. The diameter, toughness, and strength of dexamethasone PLGA implants were subsequently optimized by hot-melt extrusion to ensure batch consistency and implant performance during or after injection (Kelly et al.) [173]. NIM delivery systems consisting of topically applied triamcinolone acetonide (TA) with solid lipid NPs (TA-SLNs) and in situ gel (TA-SLN-IG) were designed to deliver the drug into the posterior segment eye (Akshaya et al.) [174]. Conjugates of TA and hydroxyl PAMAM dendrimers (D-TA) (Fig. 5) were selectively taken up by RPE and activated microglia /macrophages (mi/ma). D-TA conjugates were found to alleviate vascular leakage and inflammatory reactions by targeting “pathological” areas, tissues, and cells, suppressing inflammatory mediators and pro-angiogenic factors, and limiting macrophage infiltration. These dendrimers were also absorbed and metabolized in vitro by choroidal macrophages isolated postmortem from the eyes of patients with diabetes. Drugs to treat FNDs have also been delivered systemically by hydroxyl dendrimers [175]. In addition, PLGA microparticles [176], Au NPs [177], and arginine-glycine-aspartic acid (RGD)-polyethyleneimine (PEI) [178] have been used to deliver anti-inflammatory and anti-oxidative stress drugs such as mitomycin, resveratrol, and the oxidant salvianolic acid A (SAA) in the posterior segment of the eye for the treatment of fundus neovascularization. Administration of water-soluble hydrated fullerene C60 (C60HyFn) which has anti-oxidant, anti-inflammatory, and neuroglial protective effects, and has become a novel nanotherapeutic strategy for DR [179].

#### Molecule compounds

In addition to proteins, peptides, macromolecules, and anti-inflammatory drugs, several some molecule compounds have been shown to play roles in the normalization of the microenvironment. Nim delivery systems are therefore needed for the delivery of these molecule compounds. For example, fenofibrate, a peroxisome proliferator-activated receptor α (PPARα) agonist, was successfully encapsulated into poly (lactic-co-glycolic acid) (PLGA) NPs (Feno-NP) (Qiu et al.). The composition of the polymer was altered to optimize drug loading and long-term drug release by these Feno-NPs. These NIM delivery systems, which reduced vascular leakage, down-regulated the overexpression of VEGF and ICAM-1, and alleviated retinal neovascularization, had long-lasting therapeutic effects in patients with DR and AMD [180]. A novel sustained-release system consisting of nanoliposomes modified with the peptide APRPG (Ala-Pro-Arg-Pro-Gly) was developed to deliver triptolide (TP), which is an important immunosuppressive and anti-inflammatory compound purified from the Chinese herbal medicine Tripterygium wilfordii. The TP also exhibits anti-angiogenic activity in CNV. However, the hydrophobic nature, its half-life in tissues is relatively short., Therefore, TP-nanoliposome-APRPG (TP-nanolip-APRPG), with these NIM delivery systems targeting endothelial cells showed enhanced inhibition of TP on CNV (Lai et al.) [181].

**Fig. 6:**
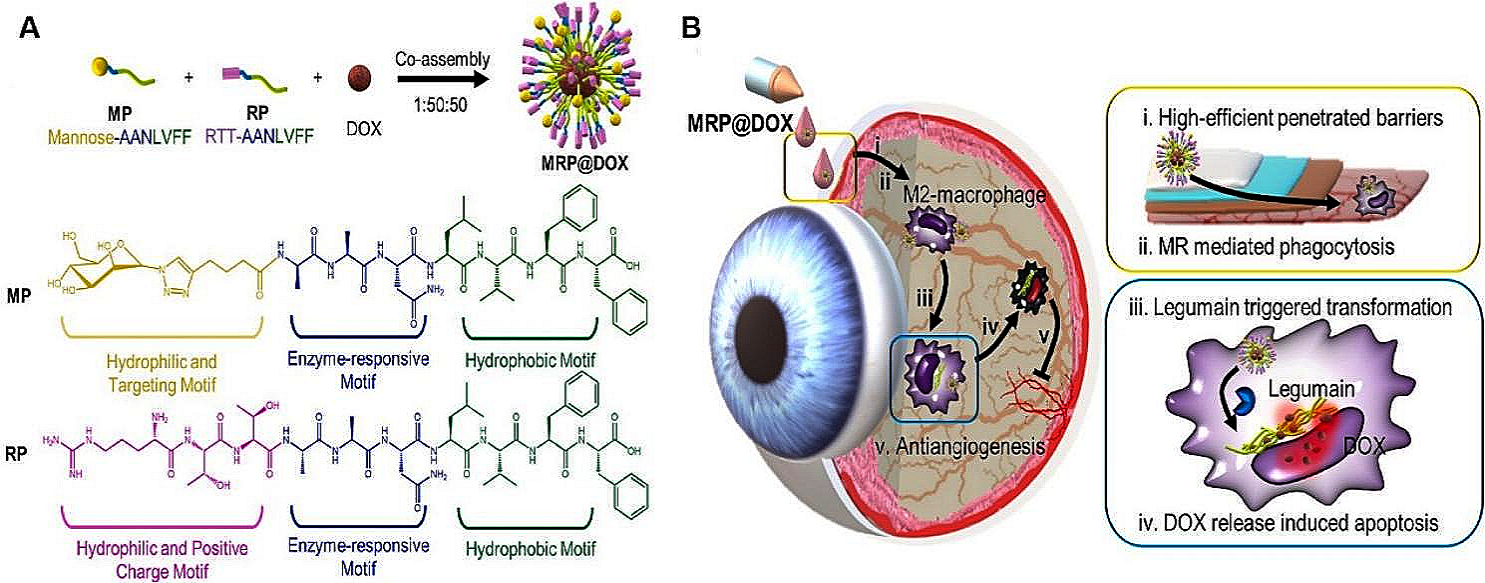
**(A)** Diagram illustrating the co-assembled components of MRP@DOX, designated as glycopeptide nanotransformers (GPNTs). **(B)** The operational steps of treatment

NIM systems have also been developed to deliver drugs non-invasively in treatment of the fundus microenvironment. For example, lysosomes carrying the cyclooxygenase (Cox) -1 and -2 inhibitors diclofenac reduced laser-induced CNV formation in mice and nonhuman primates (common marmosets) (Masamitsu et al.) [182]. Solid lipid nanoparticles (SLNs) bearing HMG Co-A reductase inhibitor Atorvastatin acid (ATS) [183], and bearing bactericidal agent isoniazid [184] in eye drops were also used to treat the fundus microenvironment in the posterior segment of the eye. Intelligent targeting light-triggered NPs based on PEG-PLA for noninvasive treatment of CNV were also developed for intravenous administration (Kohane et al.). A cell-penetrating peptide (CPP) was covalently bound to the PEG-PLA chain and then ligated to the photocleavable caging group, 7-(diethylamino) coumarin-4-yl-methyl carboxyl (DEACM) to inhibit nonspecific cellular uptake of NP-[CPP]. Irradiation with visible light at 400 nm anchored the caged CPP to the membrane of the target endothelial cells, enhancing the accumulation of NPs in the neovascular lesions. This smart nano-platform can also deliver a hypoxia-inducible factor-1 (Hif-1) antagonist doxorubicin by blocking the binding of Hif-1 to DNA to specifically treat CNV [185]. MRP@DOX are nanoparticles composed of glycopeptide (MP), cationic peptide (RP), and doxorubicin (DOX). A co-assembled MRP@DOX glycopeptide nanotransforrs (GPNTs) NIM delivery system (Fig. 6) was shown to effectively penetrate the corneal and scleral barriers, targeting M2 type macrophages and inhibiting retinal neovascularization (Li et al.). GPNTs (MRP@DOX) consist of two amphiphilic peptides: a glycopeptide (named MP) and a cationic peptide (named RP), as well as the chemotherapeutic drug: doxorubicin (DOX). The balance of proportion of DOX and two peptide amphiphiles were modularly designed will facilitate drug delivery. The workflow of using GPNTs as eye drops includes the following 5 steps. (i) Positively charged MRP@DOX enhanced penetration of the cornea and scleral barriers; (ii) Internalization into M2 macrophages, mediated by mannose receptor (MR) targeting; (iii) Legumain-induced transformation guiding lysosome escape; (iv) Enhanced retention of nanofibrous DOX, promoting apoptosis of M2 macrophages; (v) Reinforced anti-angiogenesis through elimination of M2 macrophages. These advances in GPNTs can significantly maintain the concentration of DOX and enhance its ability to induce apoptosis in M2 macrophages can help reduce M2 macrophages in the fundus microenvironment, which may contribute to blocking the formation of pathological neovascularization in a mouse model of OIR, and have potential for the non-invasive treatment of other FNDs [186].

#### Combined of anti-VEGF treatment

Inflammatory cytokines correlate positively with increased VEGF in the fundus microenvironment [187–189]. Anti-VEGF treatment, however, only temporarily reduced the abnormal levels of inflammatory cytokines [190], suggesting that beyond VEGF, abundant inflammatory cytokines and pathways other than those involving VEGF are associated with the pathophysiology of FNDs [187, 191–193]. In addition, anti-VEGF monotherapy showed limited results in the treatment of patients with refractory wet AMD [194], many of these patients responded to combination treatment with anti-inflammatory and anti-VEGF agents [195–197]. These combinations may have clinical significance in balancing the fundus microenvironment [198]. For example, PLGA Polyethyleneimine (PEI) NPs (eBev-DPPNs) loaded with bevacizumab and dexamethasone via electrostatic conjugation, were developed for the combined treatment of angiogenesis in FNDs [199]. In addition, intravitreally injected bevacizumab-dexamethasone NPs (aBev/cRGD-DPPNs), developed to target cRGD peptides, was found to inhibit CNV formation in a New Zealand white rabbit model of laser-induced CNV [200]. Lipid nano-capsules containing a novel hybrid formulation, consisting of bevacizumab (BVZ) on the surface and lipid nano-capsules containing TA in the core (BVZ-TA-LNC), were surface modified by intercalation of bifunctional polymers, and coupled to the antibody by “Click” chemistry (Formica et al.). BVZ-TA-LNC was found to significantly inhibit VEGF-induced capillary formation. This new drug delivery system allowed for the co-loading of anti-inflammatory and anti-VEGF drugs, potentially improving the treatment of inflammation and neovascularization [201]. Besides, a vascular growth inhibitor, nintedanib, and an effective antioxidant, lutein, can be assembled into nanoparticles (L/N NPs) through various non-covalent interactions. In a laser-induced CNV mouse model, minimally invasive subconjunctival administration of L/N NPs successfully inhibits angiogenesis, chronic inflammation, and oxidative stress. It achieves better therapeutic effects than standard intravitreal injection of anti-VEGF, with sustained release of both drugs for at least two months in mice (Chen et al.) [202].

**Fig. 7:**
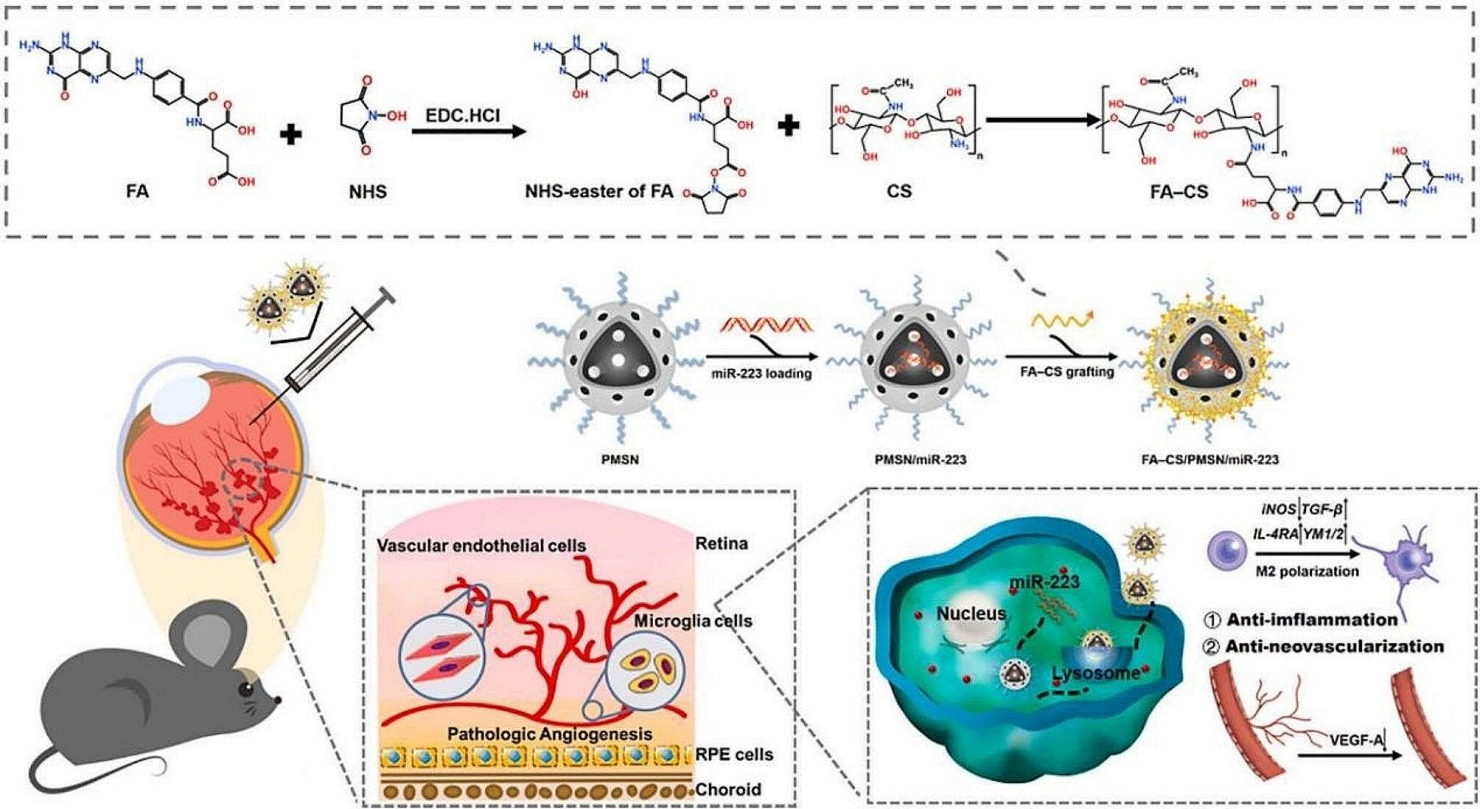
Schematic illustration of the preparation of an NIM delivery system based on miRNA-223 and its use in gene therapy to regulate anti-inflammatory and anti-angiogenesis properties in patients with retinopathy of prematurity (ROP)

However, an increasing amount of research indicates that oxidative stress and chronic inflammation play important roles in the occurrence and development of AMD [171, 203]. Macrophages in the choroidal blood flow are the main inflammatory immune cells in AMD, and the activation of macrophages is closely related to the pathogenesis of AMD [204]. In the early stages of AMD, retinal pigment epithelium (RPE) cells are damaged, leading to an increase in intracellular reactive oxygen species and recruitment of a large number of macrophages to the lesion site, where they secrete inflammatory factors, creating an inflammatory microenvironment in the RPE-retina-choroid [205]. This further activates macrophages to release inflammatory factors and matrix metalloproteinase 9 (MMP9) (forming a microenvironment with high MMP9 expression in the lesion tissue). MMP9, inflammatory factors, and VEGF form a positive feedback loop that maintains abnormal activity in the lesion site, accelerating the formation of choroidal neovascularization, and ultimately leading to irreversible damage to vision [206]. Conversely, the generation of a large number of pathological blood vessels can provide a path for driving more macrophages to infiltrate, causing persistent local inflammation, excessive pro-inflammatory signals, and pathological retinal vascular generation to mutually reinforce and cause chronic sustained disease activity, forming a vicious cycle of “oxidative stress-inflammation-MMP9-angiogenesis” [207, 208]. Furthermore, a matrix metalloproteinase peptide chain (cL) that can be degraded responsively by MMP-9, regulatory T cell (Treg) exosomes with anti-inflammatory functions (rEXS) were spatiotemporally coupled to anti-VEGF antibodies (AV) to form the rEXS-cL-aV system (Fig. 7), which was enriched in fundus neovascular lesions. This peptide chain was cleaved by MMP-9 in an inflammatory setting, releasing rEXS and aV to inhibit inflammation and VEGF activity, thus treating inflammation and suppressing VEGF activity (Tao et al.) [209]. Meanwhile, our previous work (Huang et al.) has constructed a novel multifunctional NIM delivery system, C18PGM, which can encapsulate GOQDs and minocycline within the system, effectively reducing their contact with the outside environment. By intravitreal injection, it targets activated macrophages in choroidal blood flow to achieve enrichment in target tissues and MMP9-responsive release of minocycline. When used in combination with anti-VEGF drugs, C18PGM can enhance the effect of anti-VEGF drugs by interfering with the “oxidative stress-inflammation-MMP9-angiogenesis” cascade [210].

In sum, the progression of fundus microenvironment disruption involves angiogenesis and inflammation. NIM delivery systems for combinations of anti-inflammatory and anti-VEGF agents may be a new strategy for different dosing regiments in the treatment of patients with FNDs.

### Phototherapy systems

Phototherapy utilizes light of different wavelengths to trigger photochemical or photothermal alterations in a specific tissue. The two most common forms of phototherapy are photodynamic therapy (PDT) and photothermal therapy (PTT), in which light and external or internal absorbers are employed to produce cytotoxic reactive oxygen species (ROS) or localized temperature elevation [211, 212]. The vascular effects of PDT [213–215] and its selectivity for CNV [216, 217] facilitate PDT treatment in patients with AMD. PDT can induce acute occlusion of laser-induced CNV induced by laser, inhibit the progression of CNV, and limit damage to the peripheral nerves and retina [218, 219].

#### Photothermal therapy

Photo-responsive hydrogel has been used for photo-thermal therapy and light-responsive drug delivery. Due to its temperature-dependent gelation and favorable biocompatibility [220], a number of NIM delivery systems are designed for drug control release. Like Bikram et al. used Silica-gold (SiO(2)-Au) nanoshells to release insulin [221], Hribar et al. utilized poly (b-amino ester) hydrogel to release doxorubicin [222]. A bevacizumab delivery system consisting of gold nanoparticles embedded in photo-responsive hydrogel targeting FNDs was constructed herein. Exposure to, visible light-induced gold nanoparticles converts photons into heat energy, causing the temperature of the hydrogel matrix to rise. The agarose hydrogel matrix underwent reversible softening and morphological changes to enhance the release of bevacizumab on-demand in the fundus microenvironment (Basuki et al.) [223].

#### Photodynamic therapy

Photosensitizers are molecules that undergo a photochemical reaction under specific wavelength light exposure. Photodynamic therapy (PDT) utilizes this phenomenon to exert the apoptosis of vascular endothelial cells, thereby reducing fundus leakage and neovascularization [224, 225]. As a photosensitizer, indocyanine green (ICG) can selectively target vascular lesions, improving symptoms of vascular disorders through photodynamic effects. It can also act on inflammatory tissues, inhibiting the activation of inflammatory cells and the release of inflammatory factors, thereby alleviating inflammation symptoms [226, 227]. The efficacy of ICG-loaded plasmonic Au NPs assisted combined photothermal and photodynamic therapy in promoting CNV therapy and reducing fluorescein leakage was investigated (Singh et al.). The ICG-associated enhancement of Au NPs permeability and persistence can assist in targeting therapeutic agents to the site of CNV injury [228]. Based on the complex pathological microenvironment of CNV, platinum NPs (PtNPs) and Au NPs decorated with a zeolitic imidazolate framework-8 nano platform were developed. These NPs were loaded with ICG and modified by RGD molecules (Wang et al.). The PAZIRP NPs possessed many favorable properties, including specific targeting of the CNV, precise PDT occlusion of neovascular vessels, and microenvironment regulation [4].

Verteporfin (VER) is a photosensitizer used in photodynamic therapy to eliminate abnormal blood vessels associated with conditions such as AMD. It accumulates in these abnormal vessels, and when activated by non-thermal red light with a wavelength of 693 nm in the presence of oxygen, it produces highly reactive short-lived singlet oxygen and other reactive oxygen species, causing localized damage and occlusion of the endothelium and blood vessels [229]. A novel photoactivatable NIM delivery system, Di-DAS-VER NPs, was subsequently developed to combine treatment with PDT and antiangiogenic agents (Wang et al.). This NIM delivery system first intraocularly released anti-angiogenic DAS in response to red-light irradiation of the diseased eyes, followed by the use of VER to selectively occlude neo-vessels. This red light-triggered intraocular drug release NIM delivery system enabled combined anti-angiogenic and PDT treatment of wAMD, showing that this combination treatment greatly alleviated CNV development compared with monotherapies. Moreover, treatment of CNV with Di-DAS-VER NPs showed minimum systemic and ocular toxicities [230]. Although this NIM delivery system for photodynamic therapy targeting retinal neovascular diseases is currently in the early stages of basic research and development, results to date have demonstrated the advantages and immense potential of these photodynamic NIM delivery systems for the treatment of retinal neovascular diseases. However, due to the complex structure of the eye and the different refractive indices of the intraocular media, additional research is required to evaluate the safety and effectiveness of photodynamic targeted therapy for retinal neovascular diseases as well as the feasibility of its clinical application.

### Gene therapy

Some patients may be sensitive or non-responsive to the above-described therapeutic strategies for FNDs, making it necessary to develop alternative therapeutic strategies for the long-term inhibition of pathological angiogenesis in the ocular fundus. This may be accomplished by gene therapy [231, 232], which involves the introduction of foreign genetic material, such as DNA, RNA, small interfering RNA (siRNA), microRNA (miRNA), or antisense oligonucleotides (synthetic nucleic acid sequences complementary to mRNA) into cells via viral [233] or non-viral vectors [234], to regulate or replace specific gene functions.

Early gene therapy primarily involved the introduction of a therapeutic gene to repair or replace a defective gene [235]. With the emergence and development of clustered regularly interspaced short palindromic repeats (CRISPR)/CRISPR-associated protein 9 (CRISPR/Cas9) technology, efficient gene knockout, site-specific gene insertion, and whole-genome screening have become achievable [236]. In addition, CRISPR/Cas9 can also be applied to modification of DNA/RNA epigenetic status and rewriting of histone epigenetic marks [237]. Since the first human trials using CRISPR, genome editing has been tested in the treatment of retinal diseases. Compared with anti-VEGF drug therapy or current gene therapy approaches, genome editing has unique advantages and challenges. Genome editing techniques can permanently inhibit the vascular growth pathway, thereby limiting and reducing the recurrence probability in patients with fundus neovascular diseases and providing sustained therapeutic benefits.

Viral vectors include adenovirus (ADS) [238], adeno-associated virus (AAV) [239], lentivirus, and retrovirus [240], whereas non-viral vectors generally include NPs, liposomes, dendrimers, nano-emulsions, and micelles. Most clinical trials of gene therapy for FNDs have involved two strategies, intraocular injection of a viral vector encoding anti-VEGF and siRNA targeting VEGF overexpression [241, 242]. Other, innovative and efficient NIM delivery strategies related to gene therapy have continued to emerge.

#### Non-viral vectors

Although non-viral vectors have shortcomings in gene transfer efficiency, specificity, duration of gene expression, and safety, their advantages such as lower cytotoxicity, immunogenicity, and mutagenicity make them a promising delivery system with research prospects [243, 244]. In recent years, groundbreaking progress has been made in gene therapy using non-viral vectors for the treatment of FNDs. One example is a novel siRNA-based anti-VEGF nanoballs (siVEGF NBs), in which the siRNA was sequentially encapsulated by PEI and hyaluronic acid (HA) under the influence of an electric field (NaKyung et al.). These novel siVEGF NBs have been used in a laser-induced CNV mouse model, with HA targeting CD44 receptors on the inner limiting membrane, thereby improving the targeting of NB to the sub-retinal space. Intravitreal injection of siVEGF NBs can result in the sustained inhibition of CNV weeks in mice for several weeks [245]. Similarly, intravitreal injection chitosan-hyaluronic acid nano-polyplexes, prepared using a modified ionic gelation method into rabbit eyes, could overcome the obstacles of vitreous and retina barriers and effectively reach the posterior tissues (Dinarvand et al.). Intravitreal injection of these nano-polyplexes loaded with VEGFR-2 siRNA significantly reduced the laser-induced CNV in rats [246]. APRPG-modified nanoliposomes were prepared by membrane hydration to deliver microRNA-146a-5p inhibitor, which can target abnormal endothelium, down-regulate, VEGF expression, and inhibit the cell proliferation and migration and tube and CNV formation (Li et al.) [247]. In addition to targeting VEGF genes, some genes associated with neovascularization can serve as FNDs therapeutic targets in FNDs. A cowpea chlorotic mottle virus-like particle (CCMV-VLP) was cross-linked to anti-microRNA-23 by 3,3’-Dithiobis (sulfosuccinimidyl propionate) and its ability of intravitreally injected microRNA-23 antisense oligonucleotides to selectively targeted CCMV NPs was analyzed (Chiara et al.) [248]. Our previous work (Huang et al.) constructed Folic acid–chitosan (FA–CS)-PEG-modified mesoporous silica NPs (FACS/PMSN) loaded with miR-223, this designed nanoparticle was found to reduce the retinal neovascular area in mouse models with retinopathy of prematurity by regulating retinal microglial polarization and affecting the interaction between immune cells and vascular endothelial cells. This treatment not only inhibited pathological neovascularization but normalized the microenvironment of the fundus, while not interfering with physiological neovascular development [249]. Our study highlights the potential efficacy of targeting immune cells to treat retinal neovascularization. In addition, several NIM delivery systems that deliver plasmid DNA (pDNA) have also been used to treat FNDs. For example, plasmids encoding recombinant Flt23k receptor (RGD.F1t23k.NP) and VEGF receptor (RGD.VEGF.NP) have been delivered by RGD-targeted peptide-modified NPs [250, 251]. Plasmids bearing vascular cell adhesion molecule-1 (VCAM-1) mRNA and anti-sense sequences complementary to hairpin DNA have been delivered by functionalized AuNPs (AS-VCAM-1hAuNPs) [252] and quantum dots (QD) complexed with plasmid being SOX9 DNA have been delivered by sunflower-type nanoparticles (SF-NPs) [253].In contrast to RNA interference (RNAi)-based knockdown of VEGF, some researchers use CRISPR systems for genome editing that can be modified at the DNA level to (i) persistently suppress angiogenic signals, (ii) potential disruption of extracellular and intracellular targets, and (iii) deliver to targeted cells [254]. One possible clinical strategy consists of the delivery of VEGF gene-specific Cas9 proteins and gRNAs directly to the posterior segment of the eye as ribonucleoproteins (RNPs) [255]. Subretinal injection of RNPs targeting VEGF was found to effectively reduce CNV areas in laser-induced mouse models of AMD [256]. Nano-capsules (NCs) consisting of a thin covalently-crosslinked glutathione polymer, have been used to coat Cas9 RNP complexes (Song et al.). Topical administration of these NCs to mouse RPE tissue and skeletal muscle resulted in effectively targeted gene editing capabilities [257]. It is worth noting that, non-viral vectors have lower immunotoxicity compared to viral vectors, but their NPs can trigger immune responses in vivo. The related safety issues have not been adequately evaluated and addressed [258]. Additionally, changes in physical and chemical factors during the preparation and storage process of non-viral vectors, such as pH and temperature, can affect the stability of the vectors, which is also a significant concern [259]. Since gene vectors themselves are variable and the manufacturing process involves living cells, controlling impurities and ensuring consistency between batches is also crucial. These challenges pose obstacles to the clinical translation of non-viral vectors [260].

#### Viral vectors

Although recent clinical studies have involved non-viral vectors, most clinical research studies have opted for viral vector systems due to the naturally high transduction efficiency and stable expression advantages. This choice allows for cost-effectiveness in commercial-scale production [261]. Furthermore, the maturation of gene editing technologies has also propelled research on viral vectors for gene therapy of FNDs. Lentivirus vectors (LVs) delivery of SpCas9 to the mouse retina, targeting exon 3 instead of exon 1, achieved up to 84% VEGF-A knockout in mouse RPE cells (Holmgaard et al.) [262]. Intravitreal injection of AAV9 bearing a smaller CjCas9 orthologue [263] and the type V (class II) CRISPR from Prevoltella and Francisella 1 from Lachnospiracea bacteria (LbbCpf1), which catalyzes staggered DNA cleavage [264], was found to reduce the areas of laser-induced CNV by 24% and 42%, respectively. In addition, dual AAV8 delivery of SpCas9 and gRNAs exhibited higher targeted editing rates, greater VEGF reduction, and more CNV inhibition in vivo compared with SaCas9 (Chung et al.) [265]. CasRx (RFXCAS13D) targeting of VEGF-A mRNA in the mouse retina via two guide RNAs (gRNAs) was effective in VEGF knockout and CNV inhibition (Zhou et al.) [266]. It is difficult to compare these results due to differences in CRISPR nucleases, gRNA designs, ocular delivery, and methods of quantifying efficacy. However, all of these results suggest that the reduction in VEGF protein is not in linear proportion to functional CNV inhibition and that CNV inhibition rarely exceeds 50% in rodent models, despite the degrees of genomic VEGF disruption. These findings are supported by results using clinically effective anti-VEGF drugs, such as aflibercept, which showed similar effects in animal models of CNV [264, 265]. However, a single injection of an engineered lentivirus transiently expressing nucleases and co-transmitting Cas9-mRNA and a gRNA encoding VEGF was found to effectively reduce the area of CNV without editing off-target genes or including anti-Cas9 immune responses (Cai et al.) [267]. Although VEGFR-2 has been the main target of genome-editing strategies VEGF knockdown, unlike VEGF knockdown must be targeted to endothelial cells. The dual AAV5 system, which expressed SpCas9 under the control of the endothelial cell-specific promoter ICAM2, successfully depleted VEGFR-2 and reduced angiogenesis of human retinal microvascular endothelium (HRMEC) in vitro [268]. Lentiviral vectors carrying SpCas9 targeting VEGFR2 have also been shown to have a VEGFR knockout rate > 80% and to inhibit HREC-associated VEGFR-2 > 80% in vitro [269]. In parallel, AAV1-mediated inhibition of VEGFR2 in the vitreous was observed in both OIR models and in laser-induced CNV [270]. Research on viral vectors targeting the VEGF gene has achieved remarkable success. The study of other target genes has also provided more options for gene therapy of FNDs. Hif-1 α is a major regulator of cellular hypoxic responses [271] and transcriptionally activates a variety of pro-angiogenic factors, chemokines, and receptors, including VEGF, PDGF-B, and ANG1/2. AAV9 was shown to deliver gRNAs-bearing CjCas9 targeting the Hif-1α gene to the posterior segment of the mouse eye, with a CNV inhibition rate of 20% [263], and no detectable eye toxicity or off-target effects within 14 months (Kim et al.) [272]. Interestingly, cone dysfunction was observed in the eyes that underwent VEGF, but not Hif-1α, knockout, suggesting that Hif-1α is a safer therapeutic target. Hif-1α knockout in mice using LbCpf1 reduced laser-induced CNV by up to 34% [264]. Hif-1α mediates many physiological and pathological angiogenesis pathways. Thus, permanent inhibition of Hif-1α may have other, as yet unimaginable, adverse consequences and may explain the limited use of Hif-1α antagonists, such as doxorubicin, in patients with FNDs.

Viral vectors can efficiently target not only secreted factors such as VEGF, but also intracellular targets, such as Hif-1α, as well as upstream transcription factors, regulatory elements, and downstream signaling mediators [273]. However, there is a need for greater therapeutic efficacy, including the development of novel NIM delivery systems and the maximization of cell specificity, while minimizing off-target and host immune responses.

## Conclusions and prospects

Precision, effectiveness, safety, and non-invasion have been the hallmarks of nanotechnology. Nano-medical materials have been used in a variety of eye diseases, especially in the treatment of FNDs. New NIM delivery systems can significantly inhibit fundus neovascularization, as well as have several advantages, including small particle size and material properties that are more suitable for the intraocular environment. These systems can be modified or loaded with related therapeutic agents based on their therapeutic targets and the state of FNDs progression.

To our knowledge, this review is the first comprehensive summary of the research progress of the NIM delivery systems for FNDs, including therapeutic approaches for FNDs, NIM delivery systems can cross the physiological barriers, including the BRB, in vivo and in vitro based on different therapeutic strategies. Although there have been numerous attempts to use NIM delivery systems for treating FNDs, there are still some key challenges that need to be overcome.

The eyeball has a special immune microenvironment, and ocular physiological structures may present challenges to drug delivery in FNDs. Immune imbalance of the ocular microenvironment and the breakdown of the BRB play important roles in the development of FNDs [29]. However, invasive delivery of drugs can easily cause related complications, with efficacy and safety being highly dependent on the operator’s technique and on the patient’s compliance [274]. Therefore, NIM delivery systems must be designed to prolong the half-life of the drug in the eyeball and to have good tissue permeability and histocompatibility. The focus of future research should include minimally invasive or even non-invasive delivery, with no obvious side effects or irritation of the sensitive tissues of the eye. A novel eye barrier penetration vector based on fluorocarbon chain-modified chitosan (FCS) has been developed for the delivery of large molecular ophthalmic drugs in eye drop formulations (Zhuang Liu et al.). This NIM delivery system can effectively migrate across complex eye structures to the fundus of mice and rabbits to treat of fundus tumors or CNV [275]. The NIM delivery system in the form of eye drops has great potential for the treatment of FNDs, although additional studies are required to determine its safety and efficacy.

To date, NIM delivery systems have focused on the delivery of functional groups, cells, or bioactive small molecules. These systems use nanomaterials as carriers to transport molecules such as chemicals, peptides, proteins, small molecule inhibitors, photothermal media, and chemical kinetics media. The development of nanotechnology and materials has given rise to the development of a variety of new biomaterials, coating materials, surface modification agents, and combined ligand modification agents to optimize NIM delivery systems. Intrinsic substances and the external modification of nanomaterials determine the safety, effectiveness, targeting, and biocompatibility of the NIM delivery system. Better understanding of the characteristics of various nano-delivery systems can lead to the design of NIM delivery systems that significantly improve the effectiveness and targeting ability of drugs, and reduce their toxic side effects. Precise treatment of FNDs is dependent on their pathogenesis, sites of drug action, and disease progression.

The treatment for FNDs mainly involves an anti-VEGF strategy, leading to the development of a large number of NIM delivery systems to treat FNDs. Treatment of FNDs also involves suppressing pathological vascular formation, reducing complications, preventing further damage to retinal function, and normalizing of the retinal microenvironment. For example, diabetic retinopathy (DR) is characterized by high concentrations of glucose [276] and peroxides in the microvascular area [277], as well as local hypoxia [278]. New treatment approaches can target the regulation of the retinal microenvironment by, for example, lowering blood sugar, reducing inflammation, and eliminating ROS, thereby, minimizing the progression of neovascularization and vascular leakage. AMD has long been regarded as a type of para-inflammation [279]. Age-related macular degeneration results from an imbalance between tissue damage induced by free radicals and the repair/remodeling processes of the host [280]. Para-inflammatory responses in AMD include activation of the microglia, migration beneath the retina, and activation of complement [281]. NIM delivery strategies that normalize the fundus microenvironment, remove oxidative damage, and balance pro- and anti-inflammatory responses may therefore improve treatment efficacy while reducing side effects. Retinopathy of prematurity (ROP), another significant FND, is distinguished by vaso-obliteration induced by hyperoxia, followed by delayed retinal vascularization, and pathological neovascularization triggered by hypoxia [282, 283]. In addition to regulating the fundus microenvironment, it is crucial to inhabit the pathologic, but not the developmental, angiogenesis of the fundus. A representative target is the adenosine A2A receptor, which is selectively expressed in pathological retinal neovascularization. Inhibition of A2AR through intraperitoneal administration significantly inhibits pathological vascular growth in animal models of oxygen-induced retinopathy (OIR) without affecting normal retinal vascular development. Additionally, elevated levels of A2AR have been reported in diabetic retinopathy (DR). Therefore, in the future design of nanocarrier systems for treating fundus neovascular diseases, consideration should be given to incorporating such molecules to evaluate their therapeutic activity, including their combined treatment effects with anti-VEGF therapy.

Despite ongoing research into the NIM delivery system for FNDs, clinical applicability remains limited. Different regions within human and animal tissue may differ in physiological structures and reactions, with the complexity of the tissue microenvironment increasing the difficulty of clinical application. Further research is needed to find a more optimized approach. By increasing the safety, effectiveness, targeting, and biocompatibility of active molecules under the premise of ensuring biosafety. The effects of external stimuli such as temperature, pH, pressure, and light wavelengths, on NIM delivery system complexes have been evaluated. Other biological materials may differ in their effects on the immune microenvironment of the eyeball and other biological systems. Clinical application of these NIM delivery systems also requires consideration of production costs, mass production, and the stability of the product. The development of nanobiotechnology methods for the treatment of FNDs may result in a breakthrough in treatment, with additional treatments based on NIM delivery systems benefitting patients with FNDs.

## Data Availability

No datasets were generated or analysed during the current study.

## Abbreviations

AAV: Adeno-associated virus
ADS: Adenovirus
AMD: Age-related macular degeneration
ATS: Atorvastatin acid
AV: Anti-VEGF
BBD: Box-Behnken design
BRB: Blood-retinal barrier
BVZ: Bevacizumab
CCMV: Cowpea chlorotic mottle
CCS: Core cross-linked star
CNV: Choroid neovascularization
Cox: Cyclooxygenase
CPP: Cell-penetrating peptide
CRISPR: Clustered regularly interspaced short palindromic repeats
DOX: Doxorubicin
DR: Diabetic retinopathy
ECM: Extracellular matrix
ESIONPs: Extremely small iron oxide nanoparticles
Exo: Exosomes
FCS: Fluorocarbon chain modified chitosan
FFA: Fundus fluorescein angiography
FND: Fundus neovascular diseases
GOQD: Graphene oxide quantum dots
GQD: Graphene quantum dots
HA: Hyaluronic acid
HDL: High-density lipoprotein
Hif-1: Hypoxia-inducible factor 1
HRMEC: Human retinal microvascular endothelium cells
ICG: Indocyamine green
LNPs: Lipid nanoparticles
LVs: Lentivirus vector
MAB: Monoclonal antibodies
MN: Methacrylate Nanoparticles
MMP: Matrix metalloproteinases
MP: Microparticle
MR: Mannose receptor
MVLS: Multivesicular liposomes
NBs: Nanoballs
NC: Nano-capsules
NIM: Nano-in-micro
NM: Nanoparticles-loaded microneedle
NMP: N-methyl-pyrrolidin
NPs: Nanoparticles
OCT: Optical coherence tomography
OIR: Oxygen-induced retinopathy
PCL: Polycaprolactone
PDC: Peptide-drug conjugates
PDS: Pore delivery system
PDGF: Platelet-derived growth factor
PDT: Photodynamic therapy
PEG: Polyethylene glycol
PEI: Polyethyleneimine
PLGA: Polylactide-*co*-glycolide
PMNS: PEG-modified mesoporous silica nanoparticles
PPARα: Peroxisome proliferator-activated receptor α
PPG: Polypropylene glycol
PS: Polystyrene
Pt: Platinum
PTT: Photothermal therapy
QD: Quantum dots
RBC: Red blood cell
RCEC: Retinal capillary endothelium cells
REC: Retinal endothelium cells
RNPs: Ribonucleoproteins
RNV: Retinal neovascularization
ROP: Retinopathy of prematurity
ROS: Reactive oxygen species
RPE: Retinal pigmented epithelial
RSM: Response surface methodology
RVO: Retinal vein occlusion
SAA: Salvianolic acid A
SLNs: Solid lipid nanoparticles
TA: Triamcinolone acetonide
TGF: Transforming growth factor
TP: Triptolide
VCAM-1: Vascular cell adhesion molecule-1
VEGF: Vascular endothelial growth factor
VEGFR: VEGF and its receptor
VER: Verteporfin
VLP: Virus-like particle
